# Free-standing spider silk webs of the thomisid *Saccodomus formivorus* are made of composites comprising micro- and submicron fibers

**DOI:** 10.1038/s41598-020-74469-z

**Published:** 2020-10-19

**Authors:** Christian Haynl, Jitraporn Vongsvivut, Kai R. H. Mayer, Hendrik Bargel, Vanessa J. Neubauer, Mark J. Tobin, Mark A. Elgar, Thomas Scheibel

**Affiliations:** 1 Department for Biomaterials, University of Bayreuth, Prof.-Rüdiger-Bormann-Str.1, 95447 Bayreuth, Germany; 2 ANSTO Australian Synchrotron, Infrared Microspectroscopy Beamline, Clayton, VIC 3168 Australia; 3 School of BioSciences, The University of Melbourne, Melbourne, VIC 3010 Australia; 4 University of Bayreuth, Bayreuther Zentrum für Kolloide Und Grenzflächen (BZKG), Universitätsstraße 30, 95440 Bayreuth, Germany; 5 University of Bayreuth, Bayerisches Polymerinstitut (BPI), Universitätsstraße 30, 95440 Bayreuth, Germany; 6 University of Bayreuth, Bayreuther Zentrum für Molekulare Biowissenschaften (BZMB), Universitätsstraße 30, 95440 Bayreuth, Germany; 7 University of Bayreuth, Bayreuther Materialzentrum (BayMAT), Universitätsstraße 30, 95440 Bayreuth, Germany

**Keywords:** Bioinspired materials, Biomaterials - proteins, Biochemistry, Zoology

## Abstract

Our understanding of the extraordinary mechanical and physico-chemical properties of spider silk is largely confined to the fibers produced by orb-weaving spiders, despite the diversity of foraging webs that occur across numerous spider families. Crab spiders (Thomisidae) are described as ambush predators that do not build webs, but nevertheless use silk for draglines, egg cases and assembling leaf-nests. A little-known exception is the Australian thomisid *Saccodomus formivorus*, which constructs a basket-like silk web of extraordinary dimensional stability and structural integrity that facilitates the capture of its ant prey. We examined the physical and chemical properties of this unusual web and revealed that the web threads comprise microfibers that are embedded within a biopolymeric matrix containing additionally longitudinally-oriented submicron fibers. We showed that the micro- and submicron fibers differ in their chemical composition and that the web threads show a remarkable lateral resilience compared with that of the major ampullate silk of a well-investigated orb weaver. Our novel analyses of these unusual web and silk characteristics highlight how investigations of non-model species can broaden our understanding of silks and the evolution of foraging webs.

## Introduction

The properties of orb web silks have been extensively characterized^1–6^, and they typically comprise five silk types, with two additional types found in the egg cases^7,8^. The mechanical properties vary across the silk types^7,8^ and depend, in part, on the content of crystalline and amorphous regions providing strength and extensibility^5,9–11^. All extant spiders produce silk, which may have originally been used to provide protection for the spiders and their eggs, and it is thought that foraging webs are derived from this ancestral state^12–14^ with concomitant changes in silk properties^4^. Crab spiders (Thomisidae) are typically described as ambush predators, remaining concealed in the vegetation before seizing their prey, but do not build foraging webs^15–18^. These spiders nevertheless produce draglines and attachment discs^19^, form egg cases and use silk to construct protective leaf nests^15^. The Australian thomisid *Saccodomus formivorus*^20^ is a remarkable exception, constructing a basket-like web that facilitates the capture of its ant prey^20–23^. The spiders construct their webs on low-lying shrubs that are in close proximity to either the nests or foraging trails of ants (MAE, pers obs). Foraging worker ants of several different species may be attracted to the silk of these webs, and those that venture into the basket are subsequently captured by the resident spider^23^. Clearly, the silk of the basket web of *S. formivorus* has properties that differ from those of other, conventional foraging webs. In particular, the silk must support the remarkable feature of the basket-like design: high dimensional stability that allows a free-standing web structure. Here, we describe the micro-morphology of the basket web and its structural and mechanical properties. We reveal that micro- and submicron fibers, exhibiting a distinct chemical composition, yield threads that are extraordinarily resilient against lateral loads compared with the major ampullate silk of orb webs. We further reveal that the base of the basket web contains spider eggs: this is arguably the first documented example of an elaborate spider foraging web that has evolved as an extension of the protective egg case.

## Results

### Unique morphology of the basket webs of *S. formivorus*

**Figure 1:**
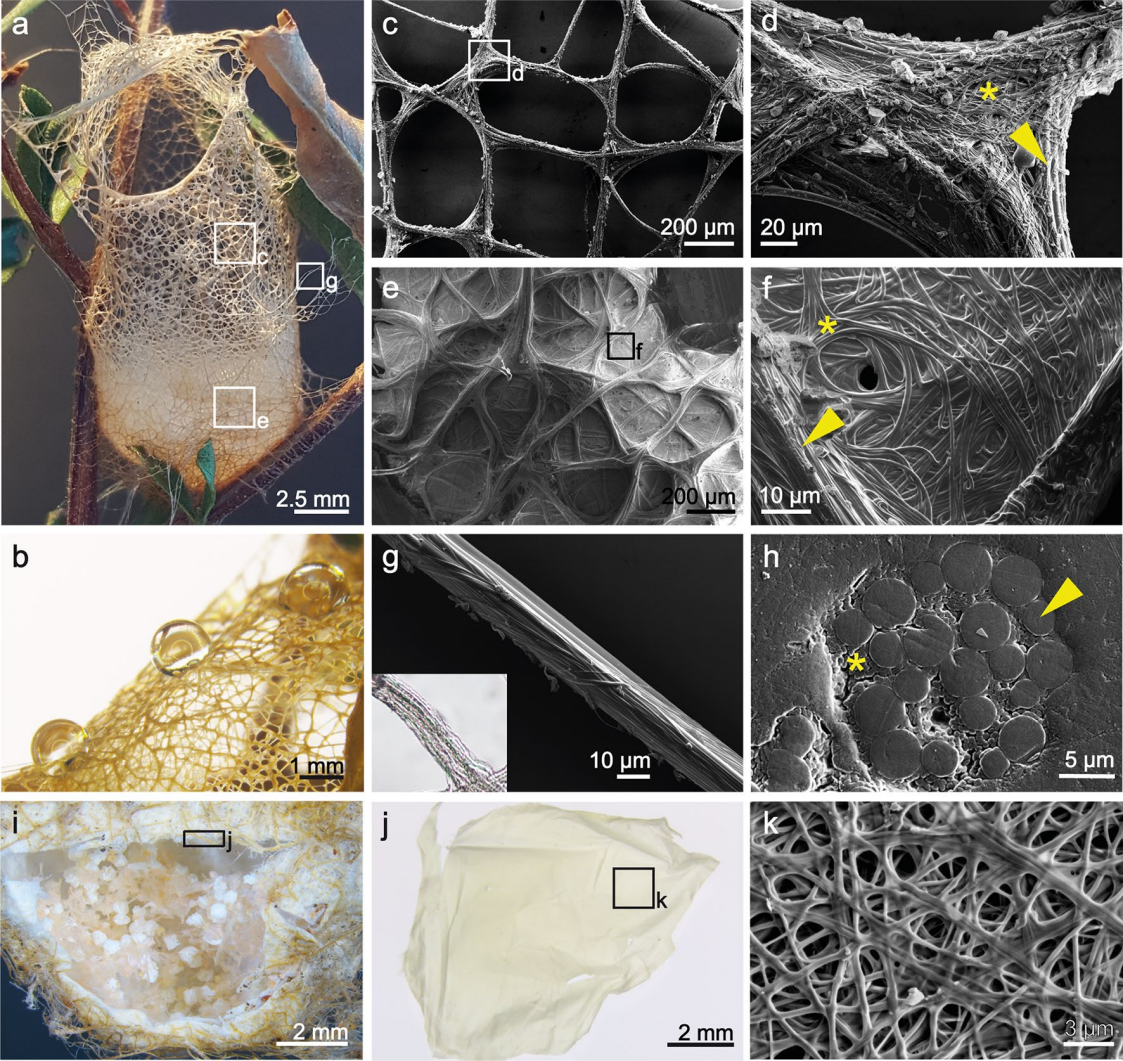
Morphological features of the silk of the web of *S. formivorus.* (**a**) Photograph of the entire basket web. (**b**) Water droplets on the web indicate high water contact angles. Scanning electron microscopy (SEM) images of (**c**) the upper and (**e**) the lower web sections originating from the zones marked in (**a**). (**d**, **f**) SEM images of marked sections in (**c**,**e**) (arrowheads indicate the presence of microfibers, asterisks show accumulated submicron fibers). (**g**) SEM image of a single protruding spider silk thread as marked in (**a**). The inset shows a light microscopic image of a protruding thread. (**h**) SEM micrograph of the thread cross-section comprising several microfibers embedded in a submicron fiber matrix (arrowhead indicates a microfiber cross-section, asterisk shows accumulated submicron fiber cross-sections). (**i**) Micrograph of the lower web section showing several hatched eggs. (**j**) Nonwoven-like sheet, which horizontally covered the eggs shown in (**i**). (**k**) SEM image of the nonwoven-like sheet as marked in (**j**).

The non-sticky webs of *S. formivorus* are cylindrical resembling a basket or “lobster pot” of 11 ± 3 mm diameter, 14 ± 4 mm depth (SD, *N* = 4) with an opening at the top that varies in size and shape. The web structure comprises crosslinked threads of varying diameters. The basket webs are firmly attached to branches and leaves by protruding threads originating throughout the web (Fig. 1a). The protruding threads resemble those that form the basket web, but are more uniform in diameter and show no crosslinks with other threads. The webs were highly water repellent (Fig. 1b), and the contact angle was estimated to be around 126° ± 11° (SD, *N* = 3).

The webs showed two distinct fiber types: microfibers of several micrometer diameter, and submicron fibers. The density of submicron fibers was lower in the upper section of the basket web (Fig. 1c), and they were distributed around the larger threads only (Fig. 1d), whereas the submicron fibers in the lower section (Fig. 1e) were also found in between the threads (Fig. 1f). As a result, the web resistance against compression increased from the upper to the lower part of the basket. At a closer look, the threads (Fig. 1g) comprised several microfibers with two to four micrometers in diameter, which were embedded in a matrix made of longitudinally oriented submicron fibers of around 400 nm in diameter (Fig. 1h).

The basket webs are not used exclusively as a retreat and foraging structure for the resident spider, as previously reported^20–23^. The base of some webs had a more pronounced submicron fiber architecture and contained hatched eggs (Fig. 1i) that were covered by a nonwoven-like sheet (Fig. 1j,k).

### Two fiber types with distinct chemical features

**Figure 2:**
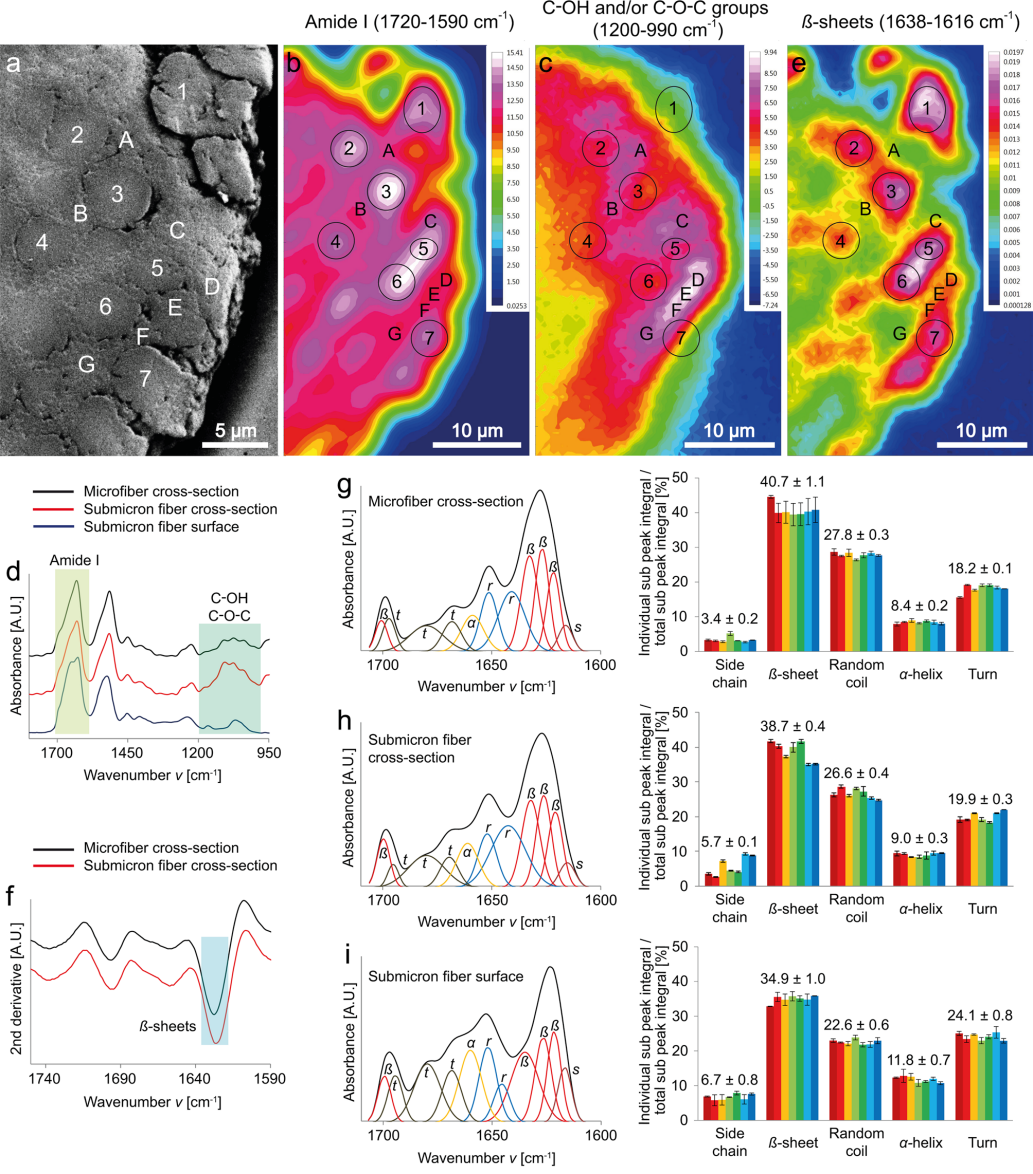
FTIR spectroscopic investigation of the silks in the basket web of *S. formivorus*. (**a**) SEM image of a thread cross-section comprising several microfibers (1–7) embedded in a submicron fiber matrix (A–G). The spatially-resolved chemical distribution (chemical map) of the thread cross-section was analyzed using synchrotron FTIR (S-FTIR) microspectroscopy, equipped with a macro ATR-FTIR device. (**b**,**c**) Chemical maps of the overall protein (i.e. amide I) distribution (1720–1590 cm^−1^) and of C–OH and/or C–O–C groups (1200–990 cm^−1^), respectively. The areas on the chemical images, indicated by number 1–7, match the location of microfibers depicted in (**a**), whereas those labelled A-G match the submicron fiber matrix. Absorption intensities correlate with the color scales and increase from blue to white. Blue-colored areas indicate no absorbance of chemicals of interest, and thereby suggesting the presence of the resin used for embedding. (**d**) Mean absorbance spectra of the microfiber cross-sections, submicron fiber cross-sections and submicron fiber surfaces. (**e**) Absorbance indicates *β*-sheet secondary protein conformation (1638–1616 cm^−1^). (**f**) Mean second derivative spectra obtained from the micro- and submicron fiber cross-sections. (**g**–**i**) Left: Fourier self-deconvolved (FSD) amide I bands of the spectra obtained from the microfiber cross-section, submicron fiber cross-section and submicron fiber surface, which are presented along with their corresponding curve fitting sub peaks (*s* side chains, *β* *β*-sheets, *r* random coils, *α* *α*-helices, *t *turns). Right: secondary protein structure proportions determined by the ratio of the respective secondary protein structure sub-peak integral to the total sub-peak integral.

We obtained spatially-resolved Fourier-transform infrared (FTIR) spectra of the thread cross-section (Fig. 2a) using synchrotron FTIR (S-FTIR) microspectroscopy, equipped with a macro attenuated total reflectance FTIR (ATR-FTIR) device, following Vongsvivut et al.^24^.

A chemical map of the protein distribution across the thread cross-section was produced based on the integrated area under the amide I band (1720–1590 cm^−1^) (Fig. 2b). Similarly, a chemical map of C–OH and/or C–O–C vibrational modes was acquired by integrating the area in the spectral range of 1200–990 cm^−1^ (Fig. 2c)^25–27^. The amide I chemical map in Fig. 2b corresponds well with the scanning electron microscopic (SEM) image of the thread cross-section (Fig. 2a), allowing the extraction of spectra on the specific areas that represented microfiber cross-sections (1–7), as well as submicron fiber cross-sections (A–G). In addition to the S-FTIR investigation, ATR-FTIR measurements of submicron fiber surfaces (i.e. of nonwoven-like sheets as illustrated in Fig. 1j,k) were conducted using a laboratory-based spectrometer equipped with a thermal IR (Globar) source. Average spectra of the micro- and submicron fiber cross-sections and of the submicron fiber surfaces are given in Fig. 2d.

Interestingly, the chemical maps showed high C–OH and/or C–O–C absorbance between the microfibers where the submicron fibers were present. In contrast, the C–OH and/or C–O–C absorbance observed on the submicron fiber surfaces was lower than that of the micro- and submicron fiber cross-sections (Fig. 2d). The second derivatives were calculated from each spectrum of the thread cross-section to identify putative crystallite regions, i.e. pleated *β*-sheets, which are one feature of spider silk fibers^9,10^. The integrated values of the respective band within the spectral range of 1638–1616 cm^−1^ were plotted to produce a chemical map allowing visualization of the spatial distribution of *β*-sheets across the thread cross-section (Fig. 2e), which clearly showed that both fiber types comprised *β*-sheets. The corresponding average second derivative spectra of the micro- and submicron fiber cross-sections are depicted in Fig. 2f, showing no significant structural differences based on their spectral features.

We used the seven extracted spectra of the microfiber cross-sections (1–7) and of the seven areas representing the submicron fiber cross-sections (A–G) for Fourier self-deconvolution (FSD) and curve fitting of the amide I bands. Therewith, we analyzed the approximate secondary protein structure proportions, which are determined by the ratio of the respective secondary protein structure sub-peak integrals to the total sub-peak integral (Fig. 2g,h). The results indicated that there are no significant differences between the cross-sections of the microfibers and submicron fibers. However, there are higher *β*-sheet and random coil contents, but less side chain, *α*-helix and turn elements in the cross-sections than on the submicron fiber surface (Fig. 2i).

### Basket threads have distinct mechanical properties

**Figure 3:**
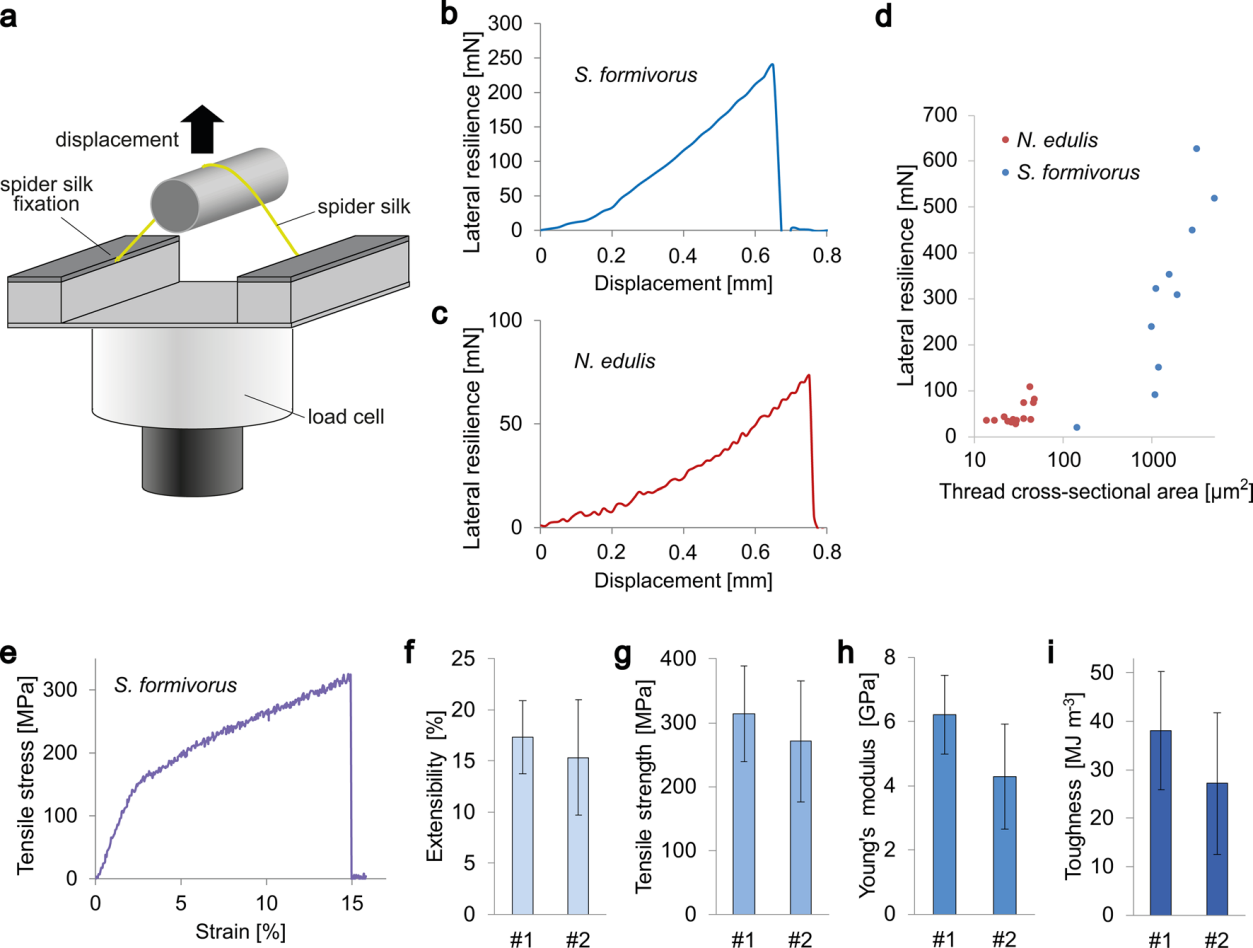
Mechanical properties of *S. formivorus* silk threads. (**a**) Schematic image of the lateral resilience test setup. (**b**) Exemplary lateral resilience–displacement plot of a thread of *S. formivorus*. (**c**) Exemplary lateral resilience–displacement plot of the double-filament major ampullate silk of the model orb weaver *N. edulis*. (**d**) Semi-logarithmic display comparing the lateral resiliencies of the silk of *N. edulis* with those of the threads of *S. formivorus*. (**e**) Representative stress–strain plot (real stress–strain) for a single thread of *S. formivorus* (according to inset g of Fig. 1a,g). (**f**–**i**) Real stress–strain data for threads originating from two webs of *S. formivorus* (#1 and #2). Error bars show standard deviation (SD).

The silk threads of *S. formivorus* have a remarkable mechanical property that has not been documented for any silk produced by spiders—namely, a resistance against lateral deformation that allows free-standing threads and webs. We determined the resiliencies of the basket threads upon lateral deformation (Fig. 3a,b). For comparison, we chose double-filament major ampullate silk of the Australian golden orb weaver *N. edulis* (Fig. 3c), which fairly reflect the mechanical properties of major ampullate silks of orb-webs^28,29^. The diameters of the examined threads of *S. formivorus* were much higher (14–80 µm) than those of the major ampullate silk of *N. edulis* (3–6 µm for single filaments). The distinct mechanical property of the basket web is revealed by comparing the resiliencies of the silk threads of *S. formivorus* that ranged from 19 to 625 mN, with that of the major ampullate double-filament silk of *N. edulis* that ranged from 25 to 107 mN (Fig. 3d).

We further analyzed the tensile properties of the threads of *S. formivorus* with a diameter range of 11–75 µm. A representative stress–strain plot and the real stress–strain data of the threads of two individual webs are given in Fig. 3e–i. In comparison to orb-webs, the tensile strength and Young’s modulus of the basket threads was not as high as those of major ampullate^7,8,28,29^ and minor ampullate silks^7,8,30^, nor did the threads exhibit the remarkable extensibility of flagelliform silks^7,8,31^. Table 1 compares the mechanical properties of the basket threads with the complete set of silk types of a model orb-web spider (here: *A. argentata* has been chosen because it is the only species with a complete set of data obtained at consistent experimental conditions^8^).

**Table 1:** Mechanical properties of threads of *S. formivorus* webs (real stress–strain data ± standard deviation (SD)) in comparison with representative orb-web spider silks of *A. argentata* (real stress–strain data ± standard error of the mean (SEM)).

Spider	Fiber type	Extensibility (%)	Tensile strength (MPa)	Young’s modulus (GPa)	Toughness (MJ m^−3^)
***S. formivorus***					
#1	Composite threads	17 ± 4	314 ± 75	6.2 ± 1.2	38 ± 12
#2		15 ± 6	271 ± 95	4.3 ± 1.6	27 ± 15
*A. argentata*^8^	Major ampullate silk	20.5 ± 0.5	1495 ± 65	8 ± 0.8	136 ± 7
	Minor ampullate silk	33 ± 3.3	923 ± 154	10.6 ± 1.2	137 ± 22
	Flagelliform silk	172 ± 5.0	534 ± 40	0.001 ± 0.0001	75 ± 6
	Aciniform silk	40.4 ± 2.4	1052 ± 120	10.4 ± 1.4	230 ± 31
	Tubuliform silk	28.6 ± 1.5	476 ± 90	11.6 ± 2.1	95 ± 17

### Basket web threads exhibit spider silk typical chemical properties

The micro- and submicron fibers within the upper and lower sections of the basket web provided good chemical resistance, as commonly observed for spider silk fibers^32,33^. In particular, the basket threads were stable for at least 7 days against chaotropic agents such as 8 M urea and 6 M guanidinium thiocyanate, the organic solvent hexafluoroisopropanol (HFIP), the ionic liquid 1-ethyl-3-methylimidazolium acetate (EMIM acetate) and 98% formic acid. In contrast, both fiber types degraded within the first 72 h after incubation in 32% hydrochloric acid, and treatment with 10 M sodium hydroxide yielded isolated fiber fragments, which disappeared after 1 week of incubation (Supplementary Fig. S1).

We further investigated the molar percentage of carbon (*C*), hydrogen (*H*), nitrogen (*N*) and sulphur (*S*) in the threads of the upper and lower sections of the basket web. Although the lower section comprised more submicron fibers than the upper section, the *CHNS* composition in both fiber types was similar (Supplementary Table S1). The outstanding dry mass of around 35% may be attributed to oxygen atoms as a major element of proteins and to inorganic materials like dust particles, which were found in between the threads (Supplementary Fig. S1).

## Discussion

The crab spider *S. formivorus* assembles micro- and submicron fibers into threads to build a basket web with an extraordinarily high dimensional stability, which provides a foraging platform to capture ants, as well as a protective refuge for the resident spider and its eggs. Although this basket web is unique across spider webs, some insects also build free-standing silken structures. For instance, the moths of the family Urodidae build cage-like, protective cocoons^34–36^, which seem to possess a similar dimensional stability compared with the webs of *S. formivorus*. Free-standing, single silk threads were further observed with lacewings, forming egg stalks made of proteins with a cross-*β* structure^37^.

Interestingly, the basket web of *S. formivorus* seems not to contain any ant deterrents such as 2-pyrrolidinone, which is found in the orb web of *Nephila antipodiana*^38^ and provides protection from ant invasion. Rather, it is likely that the basket web contains an ant-attracting compound, similar to the allomone putrescine found in the silk of orb-webs, and which enhances the capture of flies^39^.

The water repellent effect of the basket web may be partly due to the mesh-like surface of the web, which led to reduced physical interactions with water droplets, similar to the rough surface of non-wetting lotus plant leaves^40^. High hydrophobicity has also been observed in the silk used to form egg cases in other spider species^41,42^. The basket web further showed no overall stickiness, indicating the absence of aggregate-like silks, which provide the flagelliform silk of orb webs with adhesiveness for prey capturing^5,43^.

Our examinations of the microfibers and submicron fibers confirmed considerable amounts of *β*-sheets, indicating that those fibers are most likely not related to the *β*-sheet-free flagelliform silks^11^. We further compared the secondary structure contents of the microfiber and submicron fibers in the thread cross-sections and found only negligible differences. This lack of significance could be the cause of the resolution of the macro-ATR technique that is 3.2 µm^24^. Nano-IR could be an ideal technique in future investigations to shed the light into a more accurate measure of the protein secondary structure contents of submicron fibers.

There are few accounts of the silk of spider egg cases, but large tubuliform silk fibers form the outer shell and small aciniform silk fibers line the interior of those examined^5,43^. Fiber diameters of these types of silks of orb-web (araneid) spiders (*Argiope bruennichi*: 7–8 µm and 600 nm^44^, *Argiope aurantia*: 5–10 µm and 100–200 nm^45^) and tangleweb (theridiid) spiders (*Latrodectus hesperus*: 4–5 µm and 500 nm^46^) are similar to that of *S. formivorus* (2–4 µm, 400 nm). Thus, the unusual web of *S. formivorus* apparently comprises tubuliform and aciniform silks, and the foraging web has arguably evolved as an extension of the original protective egg case.

Using S-FTIR microspectroscopy, we showed that the submicron fibers within the thread cross-section had a higher content of C–OH and/or C–O–C groups compared with the microfibers, suggesting that submicron fibers in direct contact to the microfibers were modified by a C–OH and/or C–O–C-containing component (or that only the submicron fiber core comprises elevated amounts of C–OH and/or C–O–C groups, but not the submicron fiber surface). C–OH and/or C–O–C groups are basically found in saccharides^25–27^, or in proteins that result from glycosylation^47^, so these molecular compounds may be found in the threads of *S. formivorus*. Conjugated saccharides play a fundamental role in spider silk adhesiveness, facilitating attachment to substrates and cohesion of silk fibers^48^. Alternatively, C–OH absorbance might be additionally derived from conjugated amino acids such as serine, tyrosine and threonine. Longstanding research on orb web silks has deepened our understanding of their extraordinary material strength and elasticity^5,10,43^. Here, two distinct, longitudinally-oriented fiber types are assembled to form the composite threads of *S. formivorus*. In contrast to common fiber-reinforced plastics that receive their strength through incorporation of fibers into a polymer matrix^49,50^, both fiber types of the threads of *S. formivorus* may contribute to the overall tensile properties. The tensile strength and Young’s modulus of *S. formivorus* threads, assessed using tensile testing, were significantly lower than that of the major ampullate and minor ampullate silks of orb-web spiders^7,8,28–30^. In general, a fiber’s tensile strength depends on the number of material imperfections, including voids, misaligned fibrils, and free and foreign particles^51^. We discovered a high degree of cavities in the submicron fiber matrix of *S. formivorus* threads (see Fig. 1h), potentially leading to a lower tensile strength.

The remarkable property of the silk threads of *S. formivorus* is their resilience against lateral loads, which has not been previously noted in spider silk. As illustrated in Fig. 3d, the lateral resilience correlates with the thread cross-sectional area. It may be that the lateral resilience is not a function of thread diameter only, but also that the biphasic structure plays a role in the thread’s fractural mechanism. Crack propagation in the threads of *S. formivorus* may be stopped at the interface between micro- and submicron fibers similar to the situation in artificial and natural fiber-composites^52,53^, which prevents early material failure.

In terms of biomimetics, the insights of the silk threads of *S. formivorus* could be inspiring twofold. First, the threads may be used as a natural blueprint for the design of man-made rigid threads, for example for tissue engineering or textile applications. Second, the unique structure of the threads comprising two different fiber types may inspire extrusion-based processes in order to fabricate rigid composite threads in a continuous manner.

## Methods

### Spider silk web collection

We collected webs of *S. formivorus* from locations in central NSW, Australia (NSW Scientific License SL101919). The basket webs were located on low bushes located adjacent to the nest entrances or foraging trails of the meat ant, *Iridomyrmex purpureus*. No animal ethics approval was required for this study.

### Preparation of thread cross-sections

Thread cross-sections were prepared by embedding the threads of *S. formivorus* in epoxy resin (EpoFix, Struers) with a resin to hardener ratio of 15:2. After complete hardening for 3 days, the epoxy blocks were ground stepwise using silicon carbide abrasive paper discs ending up with an abrasive particle size of 2.5 µm. Polishing was carried out using MD-Dac, MD-Nap and MD-Chem discs (Struers) applied with suspensions of diamonds with 3, 1 and 0.04 µm particle sizes, respectively.

### Microscopy

Images of the webs were taken using a Leica M205C stereo microscope and a Leica Microscope DMI 3000B. For scanning electron microscopy (SEM), all samples were sputter-coated with platinum (2 nm coating) and imaged using a ZEISS Sigma VP 300.

### Contact angle measurements

In order to estimate the water contact angles on three individual webs of *S. formivorus*, a contact angle goniometer (SURFTENS Universal, OEG) was used. Contact angles were determined 1 min after droplet deposition using 0.8 ± 0.1 µl of ultrapure water.

### Chemical resistance tests

*Saccodomus formivorus* threads were incubated with either 8 M urea, 6 M guanidinium thiocyanate, hexafluoroisopropanol (HFIP), 1-ethyl-3-methylimidazolium acetate (EMIM acetate), 98% formic acid, 32% hydrochloric acid or 10 M sodium hydroxide at room temperature. After 3 and 7 days, images were taken using a Leica Microscope DMI 3000B.

### Elemental (*CHNS*) analysis

The investigation of the carbon (*C*), hydrogen (*H*), nitrogen (*N*) and sulphur (*S*) content in the basket web threads of *S. formivorus* was performed using an elemental analyzer (EA 3000 HEKAtech). Respectively 2–3 mg of the upper and lower sections of three individual webs were extracted using a sharp razor blade and transferred to tin capsules. The samples were oxidatively degraded at 1050 °C in an oxygen atmosphere, whereby complete oxidation was ensured using a tungsten trioxide catalyst. The resulting compounds were separated and quantified using a gas chromatograph with helium as carrier gas and equipped with a thermal conductivity detector. The amount of substance (mol) of carbon (*C*), hydrogen (*H*), nitrogen (*N*) and sulphur (*S*) detected in the upper and lower web sections of the basket web of *S. formivorus* were related to the total *CHNS* amount of substance yielding molar fractions (mol%).

### Infrared spectroscopy

The laboratory-based FTIR spectra of the submicron fiber surfaces originating from the webs of *S. formivorus*, i.e. of nonwoven-like sheets, were acquired using ATR-FTIR spectroscopy (Bruker TENSOR 27, Bruker Optik GmbH). The sheets were removed from the web interior using a sharp razor blade and were placed onto the sensing surface of the diamond ATR crystal before applying a pressure onto the top of the sample using an ergonomic clamp. Spectra were recorded using a spectrometer within a spectral range of 4000–600 cm^−1^ and a resolution of 4 cm^−1^ with 200 co-added scans. Background spectra of a clean ATR surface were acquired prior to each sample measurement using the same acquisition parameters. OPUS 8.0 software (Bruker Optik GmbH) was used for subsequent spectral processing and analysis.

### Synchrotron macro attenuated total reflectance Fourier-transform infrared microspectroscopy

The spatial distribution of chemical groups and of secondary protein structures in the thread cross-section were analyzed using the synchrotron FTIR (S-FTIR) microspectroscopic technique at the Australian Synchrotron IR Microspectroscopy Beamline (Victoria, Australia). The S-FTIR measurement was performed using a Bruker VERTEX V80v spectrometer coupled with a HYPERION 2000 FTIR microscope and a liquid nitrogen-cooled narrow-band mercury cadmium telluride (MCT) detector. All synchrotron FTIR spectra were recorded within a spectral range of 3900–750 cm^−1^ using 4 cm^−1^ spectral resolution. Blackman-Harris 3-Term apodisation, Mertz phase correction, and zero-filling factor of 2 were set as default acquisition parameters using OPUS 8.0 software suite (Bruker Optik GmbH). In particular, the thread cross-sections were analyzed using an in-house developed macro ATR-FTIR device equipped with a germanium (Ge) ATR hemispherical crystal (*n*_Ge_ = 4) having a 250 μm diameter sensing facet and with a 20× IR objective (NA = 0.60; Bruker Optik GmbH) following Vongsvivut et al.^24^. The spectra were not processed using ATR correction, because the spectral comparison was all made based on the spectra data that was collected using the same setup, acquisition parameters and optical configuration. As described in Vongsvivut et al.^24^, the observed spatial resolution of our synchrotron macro ATR-FTIR technique based on the parameters mentioned in the Experimental section was found to be ~ 3.20 µm. In practice, the thread cross-section, which was embedded in epoxy resin, was mounted on an aluminium disc using double-sided polyimide (Kapton) tape, and placed into the sample stage of the macro ATR-FTIR unit. First, the Ge ATR crystal was brought into the focus of the synchrotron IR beam below the 20× IR objective and, when the humidity in the nitrogen-purged enclosure surrounding the microscope stage had dropped to ~ 20%, a background spectrum was recorded in air in a non-contact mode using a projected beam size of 3.1 µm at 256 co-added scans. The thread cross-section sample was then brought into contact with the Ge ATR crystal. A rapid low-resolution overview synchrotron macro ATR-FTIR chemical map was initially acquired to determine the area and the quality of the sample contact at a 10 µm step interval using 8 co-added scans. A subsequent synchrotron macro ATR-FTIR mapping measurement was performed on specific locations within the overview map where the spectral fingerprint features of the thread cross-section were identified, using a smaller step interval of 0.5 µm and 8 co-added scans. Chemical maps were generated from the spectra by integration of the area under the specific peaks yielding spatial absorbance values as false-colored two-dimensional plots. In particular, the distribution of proteins was demonstrated by integrating the area under the amide I band (1720–1590 cm^−1^), whereas integration of the absorbance bands in the range of 1200–990 cm^−1^ showed the presence of C–OH and/or C–O–C groups. Spatial distribution of the *β*-sheet secondary protein structure was generated by calculating the second derivative of the original spectra and integrating the peak areas in the range of 1638–1616 cm^−1^. The OPUS 8.0 software (Bruker Optik GmbH) was used for subsequent spectral processing and analysis.

### Secondary protein structure content quantification

The secondary protein structure content was determined using Fourier self-deconvolution (FSD) and curve fitting of the amide I band (1720–1590 cm^−1^). Curve fitting sub peaks were applied at wavelengths 1611, 1619, 1624, 1630, 1640, 1650, 1659, 1666, 1680, 1691 and 1698 cm^−1^ to the Fourier self-deconvolved amide I band and curve fitting was performed. Secondary structures were assigned based on a protocol from Hu et al.^54^, with side chains (1605–1616 cm^−1^), *β*-sheets (1616–1622, 1622–1628, 1628–1638 and 1697–1704 cm^−1^), random coils (1638–1647 and 1647–1656 cm^−1^), *α*-helices (1656–1663 cm^−1^), and turns (1663–1671, 1671–1686 and 1686–1697 cm^−1^), which was also successfully applied in other studies^55–58^. Quantification of the secondary structure proportions was determined by the ratio of the respective sub-peak integrals to the total sub-peak integral. For data analysis, respectively 7 microfiber cross-sections, areas of submicron fiber cross-sections and submicron fiber surfaces (i.e. nonwoven-like sheets) of one individual web were analyzed. Each amide I band (of respectively 7 spectra) was deconvolved and curve fitted for three times and the mean values were calculated. For FSD curve fitting, Opus 8.0 software (Bruker Optik GmbH) was used.

### Investigation of the mechanical properties

The tensile properties of protruding (composite) threads of *S. fomivorus* webs were determined using a mechanical testing device (Bose ElectroForce 3220) equipped with a 0.49 N load cell. The dry threads were mounted onto plastic frames with a gauge length of 2 mm using a high viscosity glue (UHU Super glue) and placed into the fume hood for immediate drying. Subsequently, the threads were observed using a light microscope (Leica Microscope DMI 3000B) and their diameters were determined using Leica Application Suite X software. The threads were uniaxially loaded using an extension rate of 0.005 mm s^−1^ at a relative humidity of 30%. A total number of 27 protruding threads originating from two individual webs (#1 = 8 samples, #2 = 19 samples) were analyzed. For the calculation of real stress *σ*_*r*_ and real strain *ε*_*r*_ data, Eqs. (1) and (2) were used, whereas *σ* (engineered stress) was calculated as the force divided by the thread cross-sectional area (assumed to be circular) and *ε* (engineered strain) values were calculated as the change in thread length divided by its original length.

1 $$\sigma_{r} = \sigma \left( {{1} + \varepsilon } \right)$$

2 $$\varepsilon_{r} = {\ln}\left( {{1} + \varepsilon } \right)$$

The Young’s modulus was determined as the slope of the stress–strain plot in the linear elastic deformation range, and only regression lines were considered for data evaluation possessing a coefficient of determination > 0.9. The material toughness was assessed by integrating the stress–strain plot using Origin 8.1G. All error bars show standard deviation (SD).

Mechanical tests were conducted to analyze the lateral resilience of threads originating from the web of *S. formivorus* and of double-filament major ampullate silk of the orb weaver *Nephila edulis *(*N. edulis*). The samples were mounted onto plastic frames with a gauge length of 2 mm using a high viscosity glue (UHU Super glue) and dried immediately in the fume hood. Subsequently, the frames were horizontally fixed into a mechanical testing device (Bose ElectroForce 3220 equipped with a 2.45 N load cell) and a lateral force was applied onto the threads/fibers at a rate of 0.05 mm s^−1^ until rupture. Lateral resiliencies were investigated using 10 threads of a single web of *S. formivorus* and 14 double-filament major ampullate silks extracted from a single *N. edulis* spider. The lateral resiliencies were plotted against the cross-sectional areas of the *S. formivorus* threads and of the double-filament *N. edulis* silks. Cross-sections of the *S. formivorus* threads as well as of the *N. edulis* single filament silks were assumed to be circular.

## Supplementary information

Supplementary information.

## Data Availability

The authors declare that the data supporting our findings are available within the article and Supplementary Information.
