# Supplementary material for: Free-standing spider silk webs of the thomisid Saccodomus formivorus are made of composites comprising micro- and submicron fibers
Source: Sci Rep. 2020 Oct 19;10:17624. doi: 10.1038/s41598-020-74469-z (PMC7572385; doi:10.1038/s41598-020-74469-z)
Supplement: Supplementary file 1 — Supplementary information. [file 41598_2020_74469_MOESM1_ESM.docx]

**Supplementary Information** **for
Free-standing spider silk webs of the thomisid *Saccodomus formivorus* are made of composites comprising micro- and submicron fibers**

Christian Haynl,^1^ Jitraporn Vongsvivut,^2^ Kai R. H. Mayer,^1^ Hendrik Bargel,^1^ Vanessa J. Neubauer,^1^ Mark J. Tobin,^2^ Mark A. Elgar ^3,*^ and Thomas Scheibel^1,4,5,6,7,*^

^1^University of Bayreuth, Department for Biomaterials, Prof.-Rüdiger-Bormann-Str.1, 95447 Bayreuth, Germany
^2^ANSTO Australian Synchrotron, Infrared Microspectroscopy Beamline, Clayton, Victoria 3168, Australia
^3^The University of Melbourne, School of BioSciences, Melbourne, Victoria 3010, Australia
^4^University of Bayreuth, Bayreuther Zentrum für Kolloide und Grenzflächen (BZKG), Universitätsstraße 30, 95440 Bayreuth, Germany
^5^University of Bayreuth, Bayerisches Polymerinstitut (BPI), Universitätsstraße 30, 95440 Bayreuth, Germany
^6^University of Bayreuth, Bayreuther Zentrum für Molekulare Biowissenschaften (BZMB), Universitätsstraße 30, 95440 Bayreuth, Germany
^7^University of Bayreuth, Bayreuther Materialzentrum (BayMAT), Universitätsstraße 30, 95440 Bayreuth, Germany

^*^ To whom correspondence should be addressed: T.S. and M.A.E.
(thomas.scheibel@bm.uni-bayreuth.de, m.elgar@unimelb.edu.au)


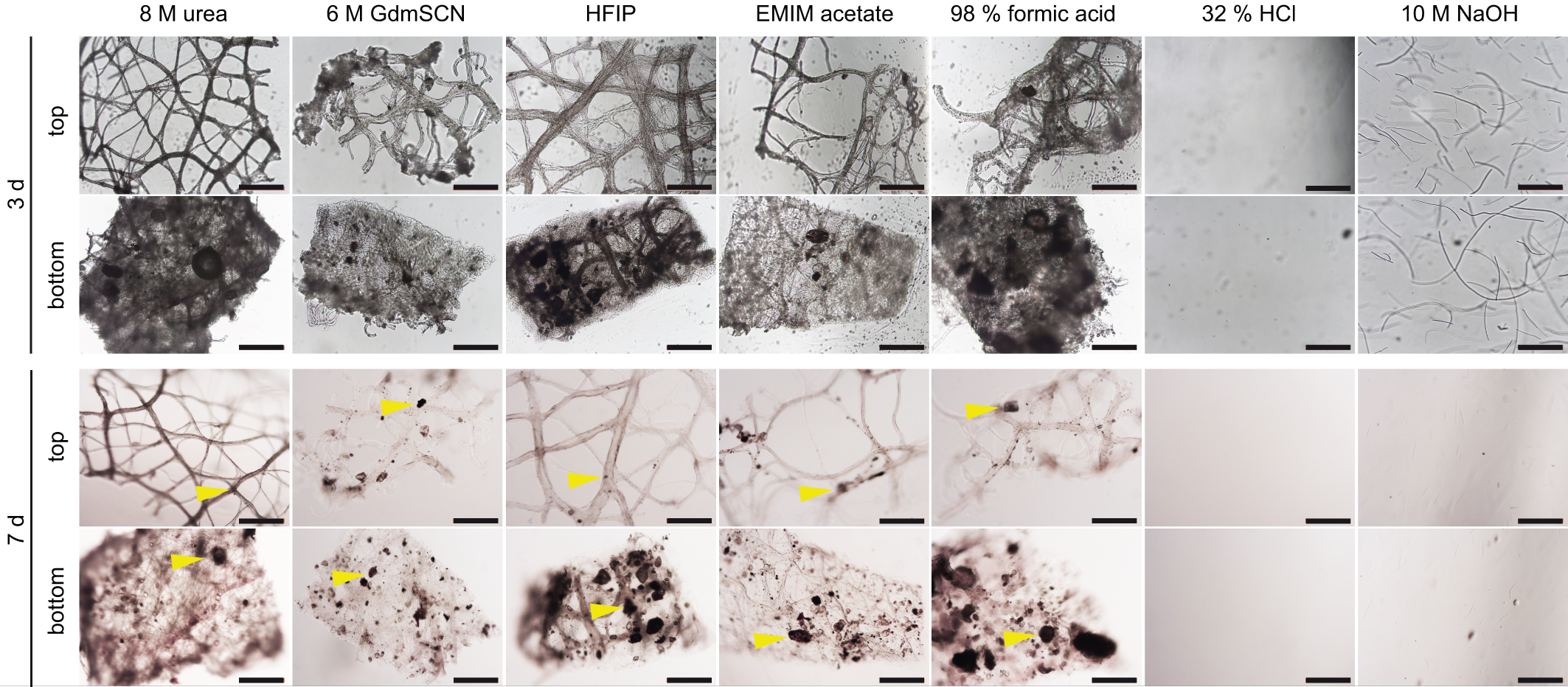


Figure S1. Chemical resistance of *S. formivorus* upper and lower web sections. Light microscopy images after 3 and 7 d incubation at room temperature in i) chaotropic agents including urea or guanidinium thiocyanate (GdmSCN), ii) hexafluoroisopropanol (HFIP), iii) 1-Ethyl-3-methylimidazolium acetate (EMIM acetate), iv) formic and hydrochloric acid and v) sodium hydroxide. Yellow arrowheads indicate dust particles. The scale bars are 500 µm.

Table S1. Molar fractions of carbon (*C*), hydrogen (*H*), nitrogen (*N*) and sulphur (*S*) found in the threads of the upper and lower sections of *S. formivorus* webs.

| **Material** | **Carbon (*C*)**  **[mol %]** | **Hydrogen (*H*)**  **[mol %]** | **Nitrogen (*N*)**  **[mol %]** | **Sulphur (*S*)**  **[mol %]** |
| --- | --- | --- | --- | --- |
| Upper section | 33.97 ± 0.38 | 56.05 ± 0.29 | 9.97 ± 0.08 | 0.01 ± 0.01 |
| Lower section | 33.78 ± 0.52 | 56.56 ± 0.80 | 9.64 ± 0.49 | 0.02 ± 0.01 |
